# *Anopheles arabiensis* and *Anopheles funestus* biting patterns in Dielmo, an area of low level exposure to malaria vectors

**DOI:** 10.1186/s12936-020-03302-9

**Published:** 2020-06-26

**Authors:** Souleymane Doucoure, Omar Thiaw, Amélé N. Wotodjo, Charles Bouganali, Nafisatou Diagne, Philippe Parola, Cheikh Sokhna

**Affiliations:** 1 VITROME, Campus International UCAD-IRD, Dakar, Sénégal; 2 Laboratoire d’Ecologie Vectorielle et Parasitaire (LEVP), Faculté des Sciences et Techniques (FST), Université Cheikh Anta Diop (UCAD), Dakar, Sénégal; 3 Aix Marseille Univ, IRD, AP-HM, SSA, VITROME, Marseille, France; 4 IHU-Méditerranée Infection, Marseille, France

**Keywords:** Malaria, Senegal, *Anopheles arabiensis*, *Anopheles funestus*, Indoor, Outdoor, Night, Morning, Season

## Abstract

**Background:**

In Dielmo, Senegal, the widespread use of long-lasting insecticidal nets has decreased both the incidence of malaria and the density of the *Anopheles* population. However, persistent low-level malaria transmission may hamper efforts to eliminate the disease. Therefore, continuous monitoring of the vector population is needed in order to improve knowledge of *Anopheles* biting behaviour and to readjust control interventions.

**Methods:**

In 2015, *Anopheles* were collected every month for a whole year and each specimen was identified using morphological and molecular techniques. The biting pattern of each species was analysed according to night (7 pm–7am) and morning (7am–11am) periods, the place of biting and the season. The ELISA CSP technique was used to assess the *Plasmodium falciparum* sporozoite rate to evaluate the entomological inoculation rate (EIR).

**Results:**

*Anopheles arabiensis* and *Anopheles funestus* sensu stricto were found to be the main vectors biting humans. Overall, the biting rate was low, at 3.84bites per night (bpn) and 1.27 bites per morning (bpm), respectively (IRR = 3.04, CI [1.84–5.00], p < 0.001). The EIR was 2.51 and 5.03 infectious bites per year during the night and morning, respectively. During the night, the *An. arabiensis* and *An. funestus* biting rate was 1.81 bpn and 1.71 bpn, respectively (IRR = 0.95, CI [0.46–1.92], p = 0.88). During the morning, their density decreased to 0.51 bpm and 0.73 bpm for *An. arabiensis* and *An. funestus*, respectively (IRR = 1.47, CI [0.58–3.71], p = 0.41). During the night and the morning, no specific trend of indoor or outdoor biting was observed in the dry and rainy season for both vectors.

**Conclusion:**

This study highlighted low level *Anopheles* nocturnal and diurnal biting and the associated risk of malaria transmission. It showed also the influence of the season on the indoor and outdoor biting pattern, indicating that the human population could be exposed all year round to a low level of *Anopheles* bites. Control programmes should increase awareness of the use of bed nets throughout the year and promote the development and implementation of complimentary tools to target *Anopheles* biting shortly after dawn when people are still indoors and outside the bed nets.

## Background

The incidence of malaria has decreased significantly over the last 15 years [1]. The decrease in incidence of the disease was made possible due to combined strategies targeting both the *Plasmodium* parasites and *Anopheles* vectors. This situation has made it possible to envisage the elimination of malaria in some endemic countries. However, malaria still remains a public health problem, as no significant progress has been made in reducing the incidence of the disease further in recent years [1]. This situation highlights the need to reinforce malaria surveillance, particularly to improve the control of *Anopheles* vectors.

Universal coverage of long-lasting insecticidal nets (LLINs) is currently the primary and most effective strategy for controlling *Anopheles* and consequently transmission of *Plasmodium* parasites [2]. The use of LLINs represents a powerful barrier against *Anopheles* mosquitoes biting and resting indoors. For this reason, the efficacy of control strategy based on LLINs use is limited when the human populations are not in bed during the period when *Anopheles* are host- seeking. Therefore, socio-environmental changes that bring human to stay outside sleeping places, where LLINs could not be used, could make a portion of the population vulnerable to *Anopheles* bites and sustain malaria transmission even at very low levels [3, 4]. On the other hand, the complex ecology of *Anopheles* may have an impact upon the effectiveness of LLINs. Indeed, it is well known that most of the efficient vectors of malaria in endemic areas have developed particular behaviour enabling them to avoid insecticide-treated nets and to feed safely on the human population [5]. This avoidance of LLINs by *Anopheles* is currently marked by significant outdoor feeding after the introduction of bed nets in some endemic areas [6, 7]. In addition to increased outdoor biting, *Anopheles* may adapt their biting time to specific periods during which the LLINs may not be used by the population. It is now observed that *Anopheles* have crepuscular and diurnal host-seeking activity which coincide, respectively, with the periods just before people go to sleep under their bed nets and just after waking up when they move outside the bed nets [8–10]. Therefore, despite a decrease in human exposure [7, 11] resulting from general coverage and a high level of bed net use, *Anopheles* behaviour could reduce the effectiveness of LLINs as people remain unprotected when mosquitoes shift their biting times or locations [9, 12]. This situation could, therefore, represent a challenge for malaria control, particularly in areas where vector controls have been implemented to eliminate the disease. Thus, close monitoring of vectors is needed in order to determine the particular biting behaviour of *Anopheles* that could put the human population at risk, despite the use of LLINs.

## Methods

### Study area

The village of Dielmo is located in the Fatick region, 280 km southeast of Dakar capital of Senegal, West Africa. In 2015, the population of Dielmo was estimated to 481 habitants distributed in 42 households. The climate is typical Sudanese-Savanna and the rainy season occurs from June/July to October/November. The annual average rainfall ranges from 400 to 900 mm and the mean temperature ranges from 22 to 35 °C. LLINs universal coverage strategy began in 2008 with three general renewal operations in 2011, 2014, 2016 and 2019. PermaNet^®^ 2.0 LLINs (active ingredient: deltamethrin) were used in 2008, 2011 and 2014 universal coverage campaigns. In 2016 campaign, a mix of PermaNet^®^ 2.0 and Olyset Net^®^ (active ingredient: permethrin) LLINs was used. In the last campaign in 2019, the coverage was done with Yorkool ^®^ (active ingredient: deltamethrin) LLINs.

### Field study and laboratory processing of mosquitoes

The human landing catch (HLC) technique was used to collect *Anopheles* mosquitoes from January 2015 through December 2015. Every month, two households were used to trap mosquitoes over three consecutive nights (7 pm to 7am) and morning (7am to 11am). These two households, 200 m apart, represent mosquitoes collection sites since the beginning of Dielmo Project in 1990 [13] until now and remained unchanged throughout the course of the study. During the night (7 pm to 7am) in each of the two sites, hourly HLC were made on two adults volunteers, one positioned inside the concession (indoor) and the other outside the concession (outdoor). In each location, indoors and outdoors, the collector was changed every 6 h. At 7 pm, in one of the collection site, the HLC were continued until 11 am, thus representing the morning collection. The collection procedure in the morning was the same as the one applied in the night.

Mosquitoes were identified morphologically using a dichotomous key described by Gilles and De Meillon [14]. In addition, a one-step PCR method using intentional mismatch primers (IMPs) was used to identify sibling species of the *Anopheles gambiae* complex [15] and the *Anopheles funestus* group [16] collected during the entire study. The crushed head and thorax was used to detect the presence of *Plasmodium falciparum* circumsporozoite protein (CSP) antigen in each *Anopheles* specimen using the ELISA-CSP technique [17].

### Rainfall data collection

During the entire study period, daily rainfall was recorded manually in Dielmo site to define a mean level of rainfall during the rainy and dry seasons.

### Data analysis

*Anopheles gambiae* complex and *An. funestus* group are the only malaria vectors involved in *Plasmodium* transmission in Dielmo. Thus, the human biting rate (HBR) and the entomological inoculation rate (EIR) were evaluated taking account only the species that belong to *An. gambiae* complex and *An. funestus* group. The HBR, which represents the density of the *Anopheles* was calculated by dividing the number of mosquitoes collected by the number of person-night/morning during the sampling period. Thus, throughout the course of the study, total *Anopheles* density during the night and morning was evaluated as well as the hourly biting rate. The EIR was obtained by multiplying the HBR by the ratio of the number of infected mosquitoes by the number of total mosquitoes screened for the presence of *P. falciparum.*

The density of the *Anopheles* was analysed according to the time of biting (night/morning), the place of biting (indoor/outdoor) and the season (dry/rainy) using a GLM (generalized linear model) with a negative binomial distribution. Analyses were performed using Stata Software, version 11.0 (College Station, Texas, USA).

## Results

### *Anopheles* species composition

A total 12 person-nights and 6 person-mornings were used to collect mosquitoes each month, representing a total of 144 person-nights and 72 person-mornings in the night and the morning, respectively, during the entire investigation. This resulted, from January to December 2015, in the collection of 680 female *Anopheles* according the following distribution: 588 (86.49%) specimens in the night and 92 (13.50%) in the morning. Morphological identification enabled us to highlight the presence of *Anopheles ziemanni, Anopheles pharoenis* and species which belong to the *An. gambiae* complex and *An. funestus* group. In order to have the precise species composition of the *Anopheles* population, molecular identification was carried out on specimen of *An. gambiae* complex and *An. funestus* group. During the night, *An. arabiensis* and *An. funestus* represented 261 (44.38%) and 247 (42%) of the collection, respectively. At the same time, *Anopheles coluzzii* and *An. gambiae* sensu stricto (*s.s.*), both of which belong to the *An. gambiae* complex, were found in the proportions of 26 (4.42%) and 20 (3.40%), respectively. However, PCR did not allow us to identify nine (1.53%) specimens of *An. gambiae* sensu lato (*s.l.*) caught during the night. *Anopheles zieman*i and *An. pharaonis* represented 16 (2.72%) and nine (1.53%), respectively, of the entire night-time collection. In the morning collection, 53 *An. funestu*s (57.60%), 37 *An. arabiensis* (40.21%) and two *An. coluzzii* (2.17%) were collected.

### *Anopheles* biting pattern

**Fig. 1:**
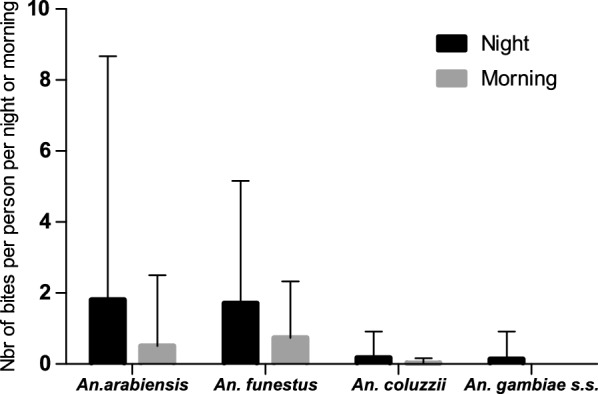
The level of human exposure to *Anopheles* bites during the night and the morning. *An. arabiensis* and *An. funestus* were the main species biting humans both at night and day with no significant difference between their biting rate (p = 0,83). *Anopheles coluzzii* and *An. gambiae s.s*. density was marginal

Overall, from 7 pm to 11 am, the biting rate was at 2.56 bites/person. The HBR was significantly different during the night and the morning with 3.84 bites per night (bpn) and 1.27 bites per morning (bpm) (Incidence rate ratio (IRR) = 3.04, 95% confidence intervals [CI] [1.84–5.00], p < 0.001), respectively. *Anopheles arabiensis* and *An. funestus* were the main species biting humans both at night and morning. During the night, *An. arabiensis* and *An. funestus* had almost the same biting rate, 1.81 bpn and 1.71 bpn, respectively (Fig. 1) (IRR = 0.95, CI [0.46–1.92], p = 0.88). During the morning, the biting rate of *An. funestus* (0.73 bpm) was slightly higher than that of *An. arabiensis* (0.51 bpm) (IRR = 1.47, CI [0.58–3.71], p = 0.41) (Fig. 1). However, during the whole study period and both during the morning and the night, there was no significant difference between the biting rate of *An. arabiensis* and *An. funestus* (IRR = 1.06 [0.61–1.82]; p = 0.83). At the same time, the biting rate the biting rate of *An. arabiensis* was significantly higher than that of *An. coluzzii* (IRR = 0.09, CI [0.03–0.24]; p < 0.001) and *An. gambiae s.s.* (IRR = 0.06, CI [0.02–0.19]; p < 0.001). The *An. coluzzii* biting rates was 0.18 bpn and 0.02 bpm, whereas *An. gambiae s.s.* biting activity was noticed only during the night (0.13 bpn) (Fig. 1).

### *Anopheles* hourly activity

**Fig. 2:**
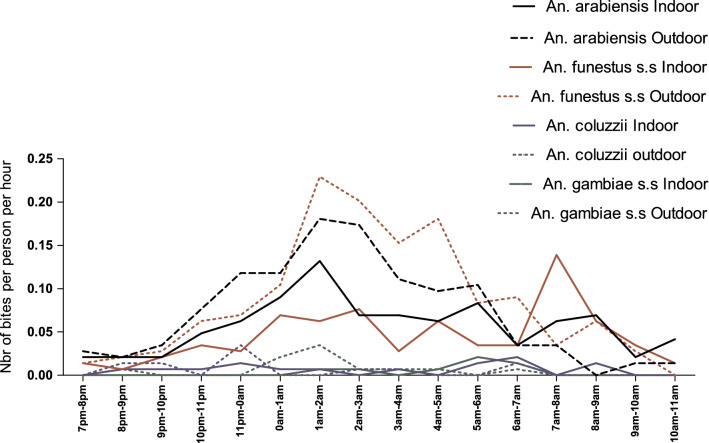
*Anopheles* hourly aggressiveness from 7 pm to 11am. Two peaks of activity involving *An. arabiensis* and *An. funestus* were observed during the first and second parts of the night. A third peak was also observed during the morning. No significant difference was observed between the activity of the first and the second part of the night (p = 0.46) and the morning (p = 0.97)

*Anopheles funestus* and *An. arabiensis* aggressiveness increased progressively throughout the first half of the night (7 pm–12 midnight) reaching 0.20 and 0.17 bites per hour, respectively. During the second part of the night (12 midnight–7am), two peaks were observed for both vectors (Fig. 2). The first peak was recorded between 1am and 2am with an HBR of 0.29 and 0.31 bites per hour for *An. funestus* and *An. arabiensis*, respectively. The second peak was observed at the end of the second part of the night, between 4am and 5am for *An. funestus* (0.24 bph) and between 5am and 6am for *An. arabiensis* (0.17 bph). The HBR of *An. coluzzii* and *An. gambiae s.s.* was very low and constant, despite weak peaks of activity occurring between 11 pm and 12 midnight, between 6am and 7am for *An. coluzzii,* and between 1am and 2am for *An. gambiae s.s.* The peak of activity in morning was observed between 7am and 8am with an HBR of 0.17 bites per hour and 0.09 bite per hour for *An. funestus s.s.* and *An. arabiensis*, respectively (Fig. 2) However, when all four species are considered, the hourly activity was not different between the first and de second part of the night (IRR = 2.50; CI [0.21–29.59]; p = 0.46), and between the first part of the night and the morning (IRR = 0.94; CI [0.03–25.53]; p = 0.97). Overall, no significant difference was observed between the night and the morning (IRR = 0.50; CI [0.03–7.46]; p = 0.62) (Fig. 2).

### *Anopheles* indoor and outdoor biting activity

**Fig. 3:**
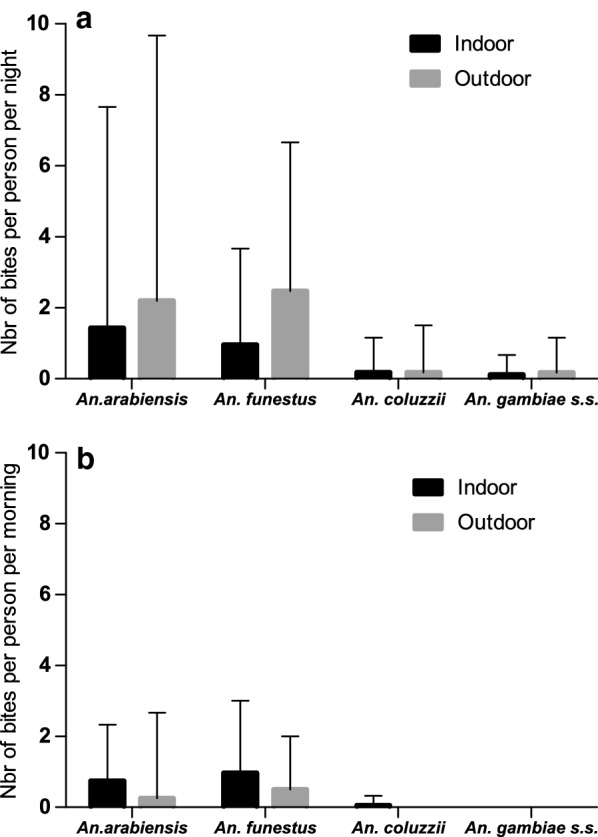
**a***Anopheles* indoor and outdoor biting rates during the night. During the night, any significant outdoor biting compared to indoor was observed for *An. arabiensis* (p = 0.404) and *An. funestus* (p = 0,08). 3b *Anopheles* indoor and outdoor biting rates during the day. During the morning, any significant outdoor biting compared to indoor was for *An. arabiensis* (p = 0.33) and *An. funestus* (p = 0.30)

The influence of the season (dry season, from December to June with 0.5 mm of cumulative rainfall; rainy season from July to November with 813.38 mm of cumulative rainfall) on *Anopheles* biting patterns was also evaluated by taking into account the place of biting (indoor/outdoor), the species and period (night/morning). Overall during the study, in the night, the HBR was higher during the rainy season (2.520 bpn) compared to the dry season (1.32 bpn) (IRR = 2.66; CI [1.47–4.80]; p = 0.001). *An. arabiensis* outdoor biting rate (2.19 bpn) was higher than that recorded indoor (1.43 bpn) despite the fact that this trend of exophagic behaviour was not significant (IRR = 1.53; CI [0.56–4.19]; p = 0.40). *An. funestus* had a HBR of 0.95 bpn and 2.47 bpn indoors and outdoors, respectively (IRR = 2.57; CI [0.90–7.37)]; p = 0.08). The *An. coluzzii* biting rate was identical indoors and outdoors (0.18 bpn); *An. gambiae s.s*. showed nearly the same biting rates indoors (0.11 bpn) and outdoors (0.16 bpn) (Fig. 3a). When the season and the biting place are combined, *An. funestus* did not displayed any significant exophagic behaviour neither in the dry season (IRR = 3.12; CI [0.68–14.28)], p = 0.14), nor in the rainy season (IRR = 2.27, CI [0.50–10.32)], p = 0.28). The same trend was observed with *An. arabiensis* in the rainy (IRR = 1.5, CI [0.35–6.29], p = 0.57) and dry season (IRR = 1.62; CI [0.34–7.58]; p = 0.54) (Table 1).

**Table 1:** Anopheline indoor and outdoor biting rate during the night according the season. Each value represents the number of bites per person per night, indoor or outdoor

	Night							
	Dry season				Rainy season			
	*An.arabiensis*	*An.funestus*	*An.coluzzii*	*An. gambiae*	*An.arabiensis*	*An.funestus*	*An.coluzzii*	*An. gambiae*
Indoor	0.40 (p = 0.54)	0.34 (p = 0.14)	0.11 (p = 0.57)	0.01 (p = 0.99)	1.02 (p = 0.57)	0.61 (p = 0.28)	0.06 (p = 0.64)	0.09 (p = 0.68)
Outdoor	0.65	1.08	0.04	0	1.54	1.38	0.13	0.16

Each value represents the number of bites per person per night, indoor or outdoor. No significant feeding behaviour according the place of biting was observed for both vector during the dry and the rainy season

During the morning, there was no significant difference (IRR = 0.91; CI [0.39–2.10]; p = 0.84) between aggressiveness during the rainy (0.51 bpm) and the dry season (0.76 bpm). The *An. arabiensis* indoor and outdoor HBR was at 0.75 bpm and 0.25 bpm, respectively (IRR = 0.33; CI [0.072–1.54]; p = 0.16) (Fig. 3b) and no significant difference was found (IRR = 0.51; CI [0.14–1.82]; p = 0.30) between the indoors (0.97 bpm) and the outdoors (0.5 bpm) regarding the aggressiveness of *An. funestus* (Fig. 3b).

During morning, in the dry season, *An. arabiensis* biting activity was only observed indoors (0.36 bpm) area while in the rainy season it was 0.38 bpd and 0.25 bpm indoors and outdoors, respectively (IRR = 0.64; CI [0.09–4.33]; p = 0.65) (Table 2). In the dry season, the indoor and outdoor aggressiveness of *An. funestus* was 0.83 bpm and 0.27 bpm (IRR = 0.33; CI [0.06–1.68]; p = 0.18), respectively. In the rainy season, its aggressiveness was at 0.13 bpm and 0.22 bpm indoors and outdoors, respectively (IRR = 1.6; CI [0.16–15.93]; p = 0.68) (Table 2). *An. coluzzii* aggressiveness was only observed indoors and only during the dry season (0.055 bpm) (Table 2).

**Table 2:** Anopheline indoor and outdoor biting rate during the morning according the season

	Morning							
	Dry season				Rainy season			
	*An .arabiensis*	*An. funestus*	*An. coluzzii*	*An. gambiae*	*An. arabiensis*	*An funestus*	*An. coluzzii*	*An. gambiae*
Indoor	0.36 -	0.83 (p = 0.18)	0.05	0	0.38 (p = 0.65)	0.13 (p = 0.68)	0	0
Outdoor	0	0.27	0	0	0,25	0,22	0	0

Each value represents the number of bites per person per morning, indoor or outdoor. No significant feeding behaviour according the place of biting was observed for both vector during the dry and the rainy season

### *Anopheles* EIR

Throughout the whole study, only two specimens were found to be using to the ELISA-CSP test: one *An. arabiensis* and one *An. funestus* collected during the night and the morning, respectively. Both positive specimens were caught outdoors, during the dry season. The EIR was, therefore, estimated to be 2.51 infectious bites per person per year during the night compared to 5.03 infectious bites per person per year during the morning.

## Discussion

In Dielmo, malaria vectors have been monitored since 1990 [18]. The results of this investigation confirm the dominance of *An. arabiensis* and *An. funestus* as the main vectors of malaria and the collapse of the *Anopheles* population that has been observed since the introduction of universal LLIN coverage [7]. In addition to *An. funestus* biting humans during daylight, as previously described [9], this study shows, for the first time, the diurnal host-seeking activity of *An. arabiensis* and *An. coluzzii*. Therefore, in Dielmo, three main malaria vectors now demonstrate diurnal host-seeking behaviour. The widespread introduction of insecticide-based control likely explains the host-seeking activity of *Anopheles* shortly before dusk and after dawn [9, 10, 19, 20]. Universal LLIN coverage has been the only strategy implemented to control the *Anopheles* vector since 2008 and has been suspected of contributing to the daytime behaviour of *An. funestus* [9]. To date, there has been no thorough investigation into the daytime behaviour of *Anopheles* vectors. It is possible that this behaviour is due to the plasticity of vectors that continue their host-seeking activity during daylight when they could not feed at night due to the use of LLINs. On the other hand, *Anopheles* mosquitoes have a specific circadian rhythm in which blood-feeding activities are preferentially performed during the night [21]. Thus, this daytime biting activity may imply a change in *Anopheles* circadian rhythms, despite the fact that experimental exposure to light can alter their biting ability [22]. Further investigations are needed to assess the basis of this diurnal biting behaviour, as it is now becoming widespread in Dielmo.

The *An. gambiae* complex and *An. funestus* groups are the only malaria vectors involved in *Plasmodium* transmission, therefore, the HBR and the EIR were evaluated accordingly. *Anopheles* morning biting activity is marginal compared to the night-time vector activity which is three times higher. Paradoxically, the level of EIR during the morning is twice as high compared to the night. The same trend was observed in Dielmo in 2011, when *An. funestus* was the sole vector biting humans during the day [9]. However, in this previous study, the level of exposure to vector bites was higher than that observed in this study, during which the incidence of the disease was very low and transmission remained seasonal [23]. Therefore, the EIR evaluated during this study should be interpreted with caution, as the level of exposure to *Anopheles* during the morning and the night is very low. Nevertheless, this study shows the importance of maintaining a continues entomological surveillance during the whole year in areas dealing with low level of exposure to vector bites and residual malaria transmission [24, 25]. However, incorporating a socio-demographic aspect into control strategies could help to provide better containment for residual transmission as human behaviour contributes greatly to malaria outbreaks in this situation [3, 26] and malaria transmission could persist at very low level even during the dry season [27].

During the night, the peak of hourly aggressiveness observed did not change compared to a previous study in Dielmo and remains confined to the second part of the night [7]. This indicates that the population could be protected while resting indoor, as the peaks observed during the night correspond to moments when the population is asleep [7], with a relatively high compliance rate in the use of LLINs [4], unlike the peak that occurs during the morning, when people are awake and remained unprotected by the LLINs. Thus, in areas striving to eliminate malaria, more attention should be paid to the possible *Anopheles* morning biting activity which essentially takes place indoors and throughout the year, which could maintain residual levels of *Plasmodium* transmission. In Dielmo, the introduction of a significant healthcare system allowing for rapid diagnosis and malaria case management [28], combined with close entomological monitoring are used to detect and/or prevent outbreaks that can result from this low level of transmission. The increase in *Anopheles* outdoor biting which was observed after the implementation of LLINs or indoor residual spraying (IRS) in endemic malaria areas [6, 7, 29], could also sustain the residual transmission of malaria. Indeed, the shift of the *Anopheles* to outdoor biting, combined with changing human behavior in Dielmo marked by increased outdoor nocturnal activity due to rural electrification could sustainably maintain residual outdoor exposure to vector bites and the occurrence of malaria outbreaks [3].Therefore, controlling outdoor exposure is the current challenge facing malaria control programmes. In this study, it appears that during the night and the morning, *An. arabiensis* and *An. funestus* showed no preference for biting outdoor or indoor regardless the season (dry or wet). It shows therefore the complexity of human exposure to *Anopheles* bites and similar attention should be paid to both the control of indoor and outdoor residual exposure when LLINs are in use [30] and whatever the season. Therefore, a more comprehensive understanding of *Anopheles* spatio-temporal dynamic and adaptive response to insecticide-treated tools is required to address this issue. In Dielmo area, previous studies showed that the introduction of LLINs induced a shift in the *Anopheles* population in favour of *An. arabiensis* which became the dominant species [31]. Furthermore, it induced a temporal and spatial structuration of the *An. arabiensis* population [32] suggesting a different subpopulation that may have a different pattern of biting. In assition, the adults *An. arabiensis* issued from a larval population carry *kdr* alleles despite the fact that this was not associated with resistance to pyrethroids [33], while no data regarding *An. funestus* is available, although its resistance to lambda-cyhalothrin has been recorded elsewhere in Senegal [34].

## Conclusion

In Dielmo, the human population is exposed to low level biting rates from three *Anopheles* vectors both during the night and at least 11 am in the morning. Despite this low level of exposure, awareness should be reinforced for LLIN use all year round. Taken as a whole, these results suggest that, in addition to the objective of developing complementary tools to control the outdoor biting of *Anopheles*, greater effort must be made to annihilate the residual daylight aggressiveness that occurs during both the dry and rainy seasons. This suggest the eventuality to assess if the current tools can be implemented to control the morning activity of *Anopheles*.

## Data Availability

The datasets used and/or analysed during the current study are available from the corresponding author on reasonable request.

## Abbreviations

Bpn: Bite per night
Bpm: Bite per morning
Bph: Bite per hour
CSP: Circumsporozoite Protein
ELISA: Enzyme-Linked Immunosorbent Assay
EIR: Entomological Inoculation Rate
HBR: Human Biting Rate
HLC: Human landing catch
LLINs: Long-lasting insecticidal nets
IMPs: Intentional mismatch primers IRS Indoor residual spraying
IRS: Indoor residual spraying
